# A comprehensive analysis of piRNAs from adult human testis and their relationship with genes and mobile elements

**DOI:** 10.1186/1471-2164-15-545

**Published:** 2014-07-01

**Authors:** Hongseok Ha, Jimin Song, Shuoguo Wang, Aurélie Kapusta, Cédric Feschotte, Kevin C Chen, Jinchuan Xing

**Affiliations:** Department of Genetics, The State University of New Jersey, Piscataway, NJ 08854 USA; Department of Genetics, Human Genetic Institute of New Jersey, The State University of New Jersey, Piscataway, NJ 08854 USA; BioMaPS Institute for Quantitative Biology, Rutgers, The State University of New Jersey, Piscataway, NJ 08854 USA; Department of Human Genetics, University of Utah School of Medicine, Salt Lake City, UT 84112 USA

**Keywords:** Human piRNA, piRNA cluster, Protein coding gene, Mobile element, High-throughput sequencing

## Abstract

**Background:**

Piwi-interacting RNAs (piRNAs) are a recently discovered class of small non-coding RNAs whose best-understood function is to repress mobile element (ME) activity in animal germline. To date, nearly all piRNA studies have been conducted in model organisms and little is known about piRNA diversity, target specificity and biological function in human.

**Results:**

Here we performed high-throughput sequencing of piRNAs from three human adult testis samples. We found that more than 81% of the ~17 million putative piRNAs mapped to ~6,000 piRNA-producing genomic clusters using a relaxed definition of clusters. A set of human protein-coding genes produces a relatively large amount of putative piRNAs from their 3’UTRs, and are significantly enriched for certain biological processes, suggestive of non-random sampling by the piRNA biogenesis machinery. Up to 16% of putative piRNAs mapped to a few hundred annotated long non-coding RNA (lncRNA) genes, suggesting that some lncRNA genes can act as piRNA precursors. Among major ME families, young families of LTR and endogenous retroviruses have a greater association with putative piRNAs than other MEs. In addition, piRNAs preferentially mapped to specific regions in the consensus sequences of several ME (sub)families and some piRNA mapping peaks showed patterns consistent with the “ping-pong” cycle of piRNA targeting and amplification.

**Conclusions:**

Overall our data provide a comprehensive analysis and improved annotation of human piRNAs in adult human testes and shed new light into the relationship of piRNAs with protein-coding genes, lncRNAs, and mobile genetic elements in human.

**Electronic supplementary material:**

The online version of this article (doi:10.1186/1471-2164-15-545) contains supplementary material, which is available to authorized users.

## Background

Piwi-interacting RNAs (piRNAs) are a recently discovered class of small non-coding RNAs that are related to, but distinct from, the better-known microRNAs. piRNAs are distinguished from microRNAs by being slightly longer (24–31 nucleotides (nt) vs. ~22 nt), mainly expressed in the germline and binding to Piwi-class as opposed to Ago-class Argonaute proteins [1–3]. Although piRNAs are abundantly expressed in both mammalian testis and ovary [4], mouse piRNA pathway mutant males are sterile but the mutant females have apparently normal oogenesis [3]. piRNAs are thought to be processed from long polycistronic RNAs transcribed from a limited number of specific loci in the genome called piRNA clusters. The genomic locations of these loci are often conserved between related species such as mouse and human [1], but the sequences of the piRNAs themselves have evolved very rapidly, differ even between closely related species such as human and chimpanzee [5].

The best understood role of piRNAs, which is based primarily on work in Drosophila and mouse, is to act as an adaptive immune system for repressing mobile elements (MEs) in the germline through a combination of post-transcriptional cleavage and DNA methylation (reviewed in [3]). This defense mechanism is thought to have deep evolutionary roots as both the piRNA machinery and ME-derived piRNAs have been identified in a wide range of metazoans, including basal lineages [6]. During this process, piRNAs are thought to alternatively cleave sense and antisense ME transcripts in a positive feedback loop called the “ping-pong cycle” [7, 8]. In mouse, different populations of piRNAs are expressed at different stages of sperm development. piRNAs produced during the pre-pachytene stage of spermatogenesis often map to MEs and a fraction of pre-pachytene piRNAs participate in the ping-pong cycle [9]. On the other hand, piRNAs produced during and after the pachytene stage are strongly depleted in ME sequences and are likely to have biological functions largely independent of ME silencing.

Recently, several lines of evidence suggest that piRNAs also play an important role in regulating endogenous gene expression (reviewed in [10]). First, in human, mouse and rat, a large fraction of piRNAs do not derive from ME regions [1] but are nonetheless under selective constraint in humans, implying that they are functionally important [5]. Second, a significant number of piRNAs are processed from mRNA transcripts in mouse, Xenopus and Drosophila, especially from 3’ untranslated regions (3’UTRs) [11–13]. The expression level of genic piRNAs does not significantly correlate with the expression level of the host mRNA, and mRNAs producing piRNAs are enriched for Gene Ontology terms different from those of genes highly expressed in the same germ cells [11]. This suggests that there exists an active mechanism to produce piRNAs from a select subset of 3’UTRs as opposed to a merely random processing of abundant mRNAs in the cell. Third, 3’UTR-derived piRNAs from the gene traffic jam in Drosophila play a role in regulating Fasciclin III and oogenesis [12]. Since most genic piRNAs are not ME-derived, these data collectively suggest that piRNAs play a role in cellular gene regulation in the germline. Nonetheless, the regulatory significance of most genic piRNAs remains unknown and their biogenesis mechanism is unclear.

Despite the emerging biological significance of piRNAs, most piRNA studies thus far have been conducted in model organisms and little is known about the abundance, diversity, origin, and function of human piRNAs. Here we performed high-throughput piRNA sequencing in three human adult testis samples. Using this dataset we analyzed the distribution of piRNAs and piRNA clusters across the human genome. We also examined their relationship with protein-coding genes, long non-coding RNA genes, and mobile genetic elements.

## Results

### Sequencing piRNAs from three human individuals

We sequenced the piRNA-enriched small RNA population of three human adult testis samples. The piRNA enrichment was performed using a periodate oxidation and β-elimination (PO treatment) protocol that is often used in piRNA studies [6, 14, 15]. Our preliminary analysis also showed that the PO treatment is very effective in eliminating the non-piRNA component of the small RNA population (Additional file 1: Figure S1).

In total, we obtained ~55 million reads from the three samples. After removing reads that match known small RNAs (e.g., miRNAs, pre-miRNAs, rRNAs, scRNAs, snRNAs, snoRNAs, srpRNAs, tRNAs, MT_tRNAs, MT_rRNAs) (Table 1, step 2), reads that cannot map to the reference genome (Table 1, step 3), and reads that are outside of the piRNA size range (Table 1, step 4), ~17 million reads were considered to be putative piRNA reads (see Table 1 and methods for data processing details). Consistent with the signature of piRNAs observed in previous studies [1, 7, 16], 75 ~ 80% of the putative piRNA reads in each individual mapped uniquely to the genome. The size distribution of the mapped reads showed a peak in the range of 26–31 nucleotides for all samples and 60 ~ 78% of the reads in that size range started with a uridine (U) in each individual. These observations suggest that our sequencing libraries are strongly enriched for piRNA sequences. Therefore we used the ~17 million putative piRNA reads (Table 1, step 4) for subsequent analyses. The final dataset contained ~6.66 million unique sequences. For simplicity, in the following section we will refer to the putative piRNA reads as piRNAs, and unique putative piRNA sequences as unique piRNAs.

**Table 1:** **Summary of the number of reads at each processing step**

Step/Sample	S9217		S7964		S7963		Total	
	Total	Unique	Total	Unique	Total	Unique	Total	Unique
Raw reads	6.85	1.89	25.30	7.41	22.91	7.57	55.05	15.07
Step 1	6.19	1.77	22.79	7.04	21.87	7.49	50.85	14.51
Step 2	3.84	1.57	14.13	6.39	13.05	6.72	31.02	13.31
Step 3	2.62	1.26	12.68	5.76	11.86	6.19	27.17	11.91
Step 4	1.47	0.82	8.83	3.55	7.13	3.22	17.44	6.66

Step 1. Remove 3’adapter sequence and remaining reads of size between 5 ~ 45 nt.

Step 2. Remove known small RNAs.

Step 3. Remove unmapped reads to the reference genome (hg19).

Step 4. Select reads of size between 26 ~ 31 nt.

Number of reads is in millions.

### piRNA clusters are conserved across individuals

It is thought that mature piRNAs originate from processing of longer RNA precursors [17]. To infer putative piRNA precursors, we mapped piRNAs to the human reference genome and identified piRNA clusters (which presumably overlap significantly with piRNA precursors) using a method similar to previous studies [1, 18]. Specifically, we slid a 5 kb window by 1 kb steps along each chromosome and counted the number of piRNAs in each window using the RPKM (Reads Per Kilobase per Million mapped piRNAs) metric (see Methods for detail).

Using a relaxed RPKM cutoff of one, we identified 6,994, 6,219, and 9,640 clusters from individual S7963, S7964, and S9217, respectively (note that our sequencing depth varied between samples). The sizes of the clusters ranged from 1 kb to 276 kb and over 80% of the putative piRNAs fell within one of the clusters. piRNAs within the new clusters have similar characteristics among individuals and with the 182 piRNA clusters identified in a previous study of human testis piRNAs [1]. Specifically, ~87% of the clusters had at least 75% of piRNAs starting with a U (Additional file 1: Figure S2A) and about three quarters of the clusters had >75% of the piRNAs on the same strand. Assuming clusters overlap significantly with precursors, this suggests that most piRNA precursors are transcribed and processed unidirectionally (Additional file 1: Figure S2B). Interestingly, although most identified clusters are novel to this study, the overall chromosomal distribution of piRNA clusters was very similar to the 182 piRNA clusters defined by [1] (Additional file 1: Figure S2C). This high degree of consistency strongly suggests that our experimental and computational methods are robust and the novel clusters we identified are bona fide piRNA clusters.

**Figure 1:**
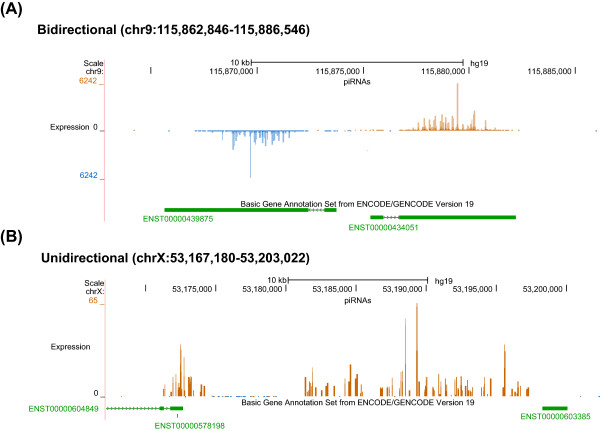
**Two examples of piRNA clusters. (A)** Bidirectional; **(B)** Unidirectional. The genomic location of the locus, piRNA mapping density, and GENCODE gene annotation are shown. piRNAs mapped to the sense and antisense strand of the reference genome are shown in brown and blue, respectively. The mapping density plot is generated by UCSC genome browser Custom Tracks tool. For gene annotation, exonic regions are shown as solid boxes, while non-exonic regions are shown as thin lines, with arrows indicating the direction of the gene.

Although the expression level of the clusters among samples varied, the piRNA clusters we identified were largely consistent among individuals. Therefore, we pooled the data from all three individuals for cluster identification. Using the pooled data we identified 6,250 clusters (cutoff RPKM = 1) and ~90% of these clusters were found in any one of the three individuals. This result suggests that piRNA clusters are largely conserved across human individuals and the observed variation between individuals could be attributed to differences in sequencing depth. The identified piRNA clusters includes more than 98% of the 182 known piRNA clusters [1], and our much higher sequencing depth allowed us to identify many more clusters expressed at lower levels. Figure 1 shows two piRNA cluster examples.

To determine the effect of the RPKM cutoff on the cluster identification, we applied multiple cutoffs. As expected, fewer piRNA clusters were identified with more stringent cutoffs (Additional file 2: Table S1). At a RPKM cutoff of 10 (roughly comparable to that used in [1]), we found 204 clusters, which is very close to the 182 clusters identified in the study [1]. One hundred and fourteen of the 182 clusters (~63%) were present in our data set (Additional file 2: Table S1). Because the cutoffs used in the literature are arbitrary and were defined when the sequencing depth was much lower than it is now, we decided to provide a list of all piRNA cluster candidates that passed the relaxed cluster definition (RPKM = 1). The RPKM value for each piRNA cluster candidate is available for researchers who wish to use a different cutoff (Additional file 2: Table S2).

### The majority of human genic piRNAs are derived from 3’UTRs

Previous studies suggested that mRNA/3’UTR-directed piRNA generation could be a major mechanism for the primary piRNA biogenesis in mouse and Drosophila [11]. We examined the piRNAs mapped to protein-coding genes to determine the potential role of protein-coding genes in piRNA biogenesis in human. We found 1,656,819 piRNAs (9.5% of the total piRNAs) mapped to the exonic regions of genes on the sense strand, while only 217,546 piRNAs (1.25% of the total putative piRNAs) mapped to exonic regions on the reverse strand. The vast majority of these piRNAs are unique: 1,176,700 and 172,593 unique piRNAs (17.67%, 2.59% of the total unique piRNAs) mapped to the exonic regions of genes on the sense and antisense strand, respectively. We define piRNAs that map to exonic regions of protein-coding genes on the sense strand as “genic piRNAs”. The genic piRNAs show strong Uridine enrichment at their first position (79.6%). The vast majority of the genic piRNAs (95.5%) mapped uniquely to the genome and are depleted in ME sequences: while ~5.7% of genic regions are ME-derived, less than 2% of genic piRNAs are ME-derived. Compared to genic piRNAs in mouse, our data suggests humans have a higher proportion of genic piRNAs: only ~2% of piRNAs are genic in mouse adult testis [11]. This difference is consistent with previous results where 11% of human piRNAs and 3% of mouse piRNAs map to protein-coding genes, respectively [1].

**Figure 2:**
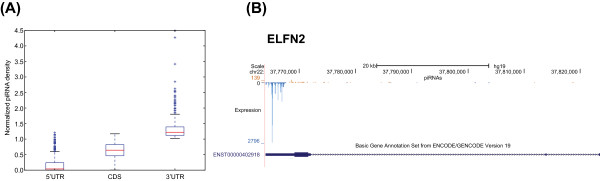
**piRNA mapping pattern in protein-coding genes. (A)** piRNA densities in the 5’UTR, CDS, and 3’UTR regions of 3’UTR piRNAs enriched genes. piRNA density in each region is shown in boxplot. For visual clarity, genes with normalized piRNA enrichment > 5 are not shown. **(B)** piRNA mapping density in 3’UTR piRNA enriched gene ELFN2 (Extracellular Leucine-rich repeat and Fibronectin type III domain containing 2). The genomic location of the locus, piRNA mapping density, and ENCODE gene annotation are shown. piRNAs mapped to the sense and antisense strand of the reference genome are shown in brown and blue, respectively. For gene annotation, exonic regions are shown as solid blue boxes, while non-exonic regions are shown as thin lines, with arrows indicating the direction of the gene. piRNAs predominantly mapped to the 3’UTR of ELFN2 in the direction of the gene (i.e., antisense to the reference genome).

It is known that genic piRNAs tend to be derived from 3’UTRs in other animals [11]. To examine this pattern in human, we calculated the piRNA enrichment in each of the three genic regions, 5’UTR, CDS, and 3’UTR. For genes with at least 300 piRNAs, we found a significant enrichment of piRNAs in 3’UTRs, as compared with CDSs and 5’UTRs (Figure 2A, Mann–Whitney U test: p < 4.3×10^−9^ for CDS and p < 1.9×10^−173^ for 5’UTRs). Among all genes containing genic piRNAs, 510 (55%) have the highest number of piRNAs in their 3’UTRs (Additional file 2: Table S3) and we call these genes “3’UTR piRNA enriched genes”. The percentage of first Uridine among piRNAs within 3’UTR piRNA enriched genes is higher than all genic piRNAs (82.5% vs 79.6%), and the percentage is even higher for piRNAs within the 3’UTRs of these genes (83.7%). This result strongly suggests that the vast majority of these putative piRNAs are processed by piRNA biogenesis machinery. One example of the piRNA mapping pattern in a 3’UTR piRNA enriched genes ELFN2 is shown in Figure 2B. One plausible model for explaining the presence of piRNA-enriched genes is that the genic piRNA biogenesis machinery processes mRNAs from the transcriptome in proportion to their cellular abundance. To test this hypothesis, we determined the correlation between piRNA abundance and gene expression level. We found a weak positive correlation between the number of 3’UTR-derived piRNAs and the gene expression level for all genes expressed in testis using previously published data [19] (Pearson’s r: 0.05, p < 8×10^−9^). However, the most highly expressed genes in testis [19] did not produce proportionally larger number of piRNAs. The median number of 3’UTR piRNAs in the top 10% highly expressed genes is just 8.6, whereas that in the 510 genes enriched for 3’UTR piRNAs is 459.7. Thus, it is more likely that an active molecular mechanism is responsible for genic piRNA biogenesis from a subset of expressed genes in testes, and preferentially from their 3’UTRs.

Next we performed Gene Ontology (GO) term analysis for the 510 genes enriched for 3’UTR piRNAs compared to the 510 most highly expressed genes in testes that are not associated with piRNAs. We found that the GO terms over-represented in the 3’UTR piRNA enriched genes were very different from those in the highly expressed genes (Additional file 2: Table S4). Genes enriched in piRNAs in their 3’UTR were more likely to be involved in chromatin modification (GO:0016568, Bonferroni corrected p < 5.1×10^−4^) and regulation of cellular metabolic process (GO:0031323, Bonferroni corrected p < 3.0 ×10^−3^), whereas highly expressed genes not associated with piRNAs were involved in other biological processes such as mRNA metabolic process (GO:0016071, Bonferroni corrected p < 3.3×10^−15^) and spermatogenesis (GO:0007283, Bonferroni corrected p < 1.1×10^−4^).

The number of 3’UTR-derived piRNAs showed significant correlation with 3’UTR length (Pearson’s r: 0.19, p < 6.3×10^−106^). Indeed, genes enriched in piRNAs in their 3’UTR tend to have much longer 3’UTR length than all other genes (median 3’UTR length: 3300.5 > 890, Mann–Whitney U test: p < 1.4×10^−156^). If piRNAs are randomly produced from the 3’UTR of any mRNA, we would expect that the piRNA producing genes will fall in the same category as the genes with the longest 3’UTRs in the GO term analysis. Therefore, we performed GO term analysis for the 510 genes that have the longest 3’UTR and are not associated with piRNAs (Additional file 2: Table S4). Genes with the longest 3’UTRs shared enrichment with piRNA producing genes in some categories, such as metabolic process (GO:0031323) and protein modification process (GO:0036211). However, other categories, including chromatin modification (GO:0016568) and chromatin organization (GO:0006325) were specific to piRNA producing genes. Therefore, our results suggest that although piRNA biogenesis pathways tend to produce piRNAs from genes that have long 3’UTRs, the genes are not randomly selected and are enriched for specific cellular functions.

### piRNA-producing genes are conserved across species

To determine if the 3’UTR piRNA enriched genes are conserved across species, we identified the human orthologs of the 3’UTR piRNA enriched genes in mouse and Drosophila from [11]. We found that the human orthologous genes of mouse 3’UTR piRNA enriched genes have significantly more 3’UTR-derived piRNAs than all other genes (Mann–Whitney U test: p < 1.2×10^−208^ for mouse 10 dpp, p < 3.7×10^−98^ for mouse adult testis; Additional file 1: Figure S3A, S4A). Even when controlling for 3’UTR length, normalized piRNA enrichment in 3’UTR, and gene expression level, 3’UTR piRNA producing genes are still highly conserved between the two species (Additional file 1: Figure S3B-D, S4B-D). Furthermore, human genes orthologous to Drosophila 3’UTR-enriched genes also have significantly more 3’UTR piRNAs than all other genes (Mann–Whitney U test: p < 5.79×10^−6^, Additional file 1: Figure S5).

Performing the analysis on the gene-by-gene basis, 3’UTR enriched genes in human significantly overlap with genes homologous to 3’UTR enriched genes in mouse but not with Drosophila (hypergeometric test: mouse 10dpp: p < 2.0×10^−99^, mouse adult: p < 3.3×10^−34^, Drosophila: p > 0.096). Using piRNA precursors identified in mouse testes [13], we found a similarly significant level of overlap (hypergeometric test: 2.0×10^−30^). Taken together, these data indicate that piRNA-producing genes are conserved between human and mouse but not between these mammals and Drosophila.

### Some long non-coding RNAs may act as primary piRNA transcripts

Long non-coding RNAs (lncRNAs) are a diverse class of RNAs >200 nucleotide long with no apparent coding capacity but often expressed in a cell-type and developmental stage-specific pattern [20]. Despite the existence of a few well-studied lncRNAs such as Xist, the biological function of most lncRNAs is still unknown. We hypothesize that some annotated lncRNA genes might be piRNA precursors.

**Figure 3:**
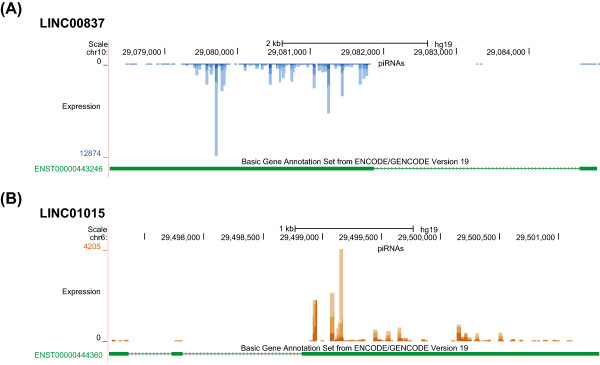
**Two examples of lncRNAs that could act as piRNA precursors. (A)** LINC00837; **(B)** LINC01015. The genomic location of the locus, piRNA mapping density, and ENCODE gene annotation are shown. piRNAs mapped to the sense and antisense strand of the reference genome are shown in brown and blue, respectively. For gene annotation, exonic regions are shown as solid green boxes, while non-exonic regions are shown as thin lines, with arrows indicating the direction of the gene.

To test this hypothesis, we examined piRNA expression levels in 15,013 previously annotated lncRNA genes. To increase the specificity of the analysis, we only included piRNAs that mapped to the sense strand of lncRNA exons. Under this stringent condition, 89 to 487 lncRNA loci showed appreciable amount of piRNA expression, depending on the cutoff we used (Table 2, the complete list of the 487 lncRNA loci is provided in Additional file 2: Table S5). Two examples of the piRNA mapping patterns in lncRNAs are shown in Figure 3. For 131 lncRNA loci, their exonic regions showed piRNA expression level > =10 RPKM and piRNAs mapped to >50% of the exonic regions. This enrichment is highly significant: the exonic regions of the 131 lncRNA genes cover only 0.016% of the whole genome but derive 15.7% of the total putative piRNAs, 965 times more than expected by chance (χ^2^ test, p < 10^−99^). The piRNA expression is also highly enriched in exons among the 131 lncRNAs: for the 114 lncRNAs that contain both exonic and non-exonic regions, the piRNA expression in exonic regions is significantly higher than that in their non-exonic regions (RPKM median: 26.88 vs. 1.71; Mann–Whitney U Test: p < 2.1x10^−20^). Overall, these results suggest that some annotated lncRNA genes might serve as precursor transcripts of piRNAs, and up to 16% of piRNAs can be produced from these loci.

**Table 2:** **Candidate piRNA-producing lncRNAs**

RPKM cutoff	Coverage cutoff	No. of lncRNAs producing piRNA	Total % of piRNAs overlapping lncRNAs	%1U in piRNAs overlapping lncRNAs
1	50%	487	16.1%	84.7%
3	50%	331	16.0%	84.7%
10	50%	131	15.7%	84.7%
1	70%	185	14.8%	84.6%
3	70%	153	14.8%	84.6%
10	70%	89	14.7%	84.6%

Recently it became apparent that lncRNAs are enriched for particular classes of MEs embedded in their exons [21, 22]. Given the strong overlap we observed between piRNAs and mature lncRNAs, we examined the ME content of the 487 lncRNAs in Table S5 (referred to as “pi-lncRNAs”). As a whole, the ME content of pi-lncRNAs (34.1% of exonic sequence) does not depart from that of other lncRNAs (39.9% of exonic sequence) (Additional file 1: Figure S6). As in the rest of the human genome, the ME content of lncRNAs is numerically dominated by non-LTR elements (SINEs and LINEs) (Additional file 1: Figure S7A). Even though this bias is still visible for pi-lncRNAs, LTR/ERV elements are more abundant (Additional file 1: Figure S6 and S7A). Moreover, the most statistically enriched ME families in pi-lncRNAs are predominantly of the LTR/ERV class (Additional file 1: Figure S7B), as previously documented for all lncRNAs [21, 22].

### piRNAs mapped to mobile elements

The best understood function of piRNAs is regulating MEs in several species [3]. To assess the potential role of piRNA in ME control in human, we examined the piRNAs that mapped to known MEs in human. Overall 22% of piRNAs mapped to MEs in the reference genome. This fraction was similar to previous studies of mouse (17%) piRNAs but less than Drosophila piRNAs (45%) [1, 7], even though MEs occupy more DNA in the human genome (about 50%, hg19) than in Drosophila (~27%, dm3).

**Figure 4:**
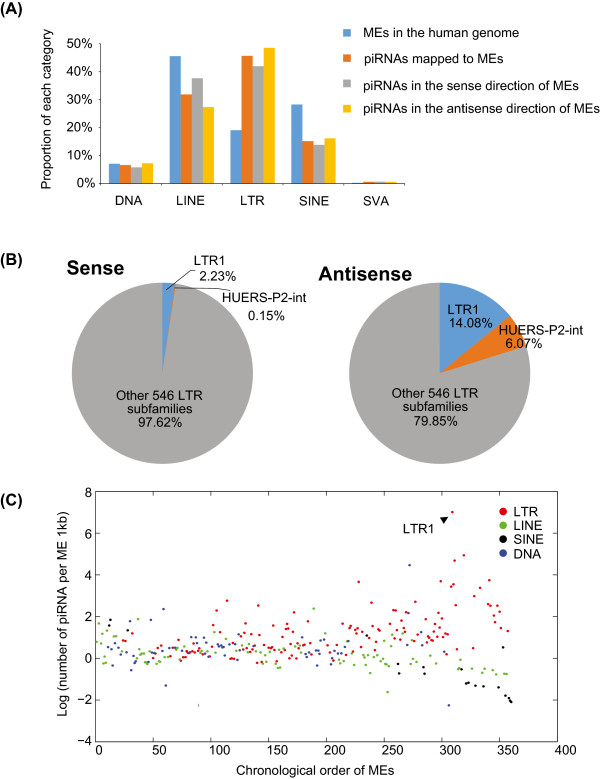
**piRNA mapped to different ME subfamilies. (A)** Proportion of piRNAs mapped to different ME families. ME families DNA, LINE, LTR, SINE, and SVA are shown. The relative proportion of the ME families in the reference genome is shown for reference. **(B)** Proportion of piRNAs mapped to different LTR subfamilies. Left and right charts show the proportion of piRNAs mapped to sense and antisense strand, respectively. **(C)** The normalized number of piRNAs mapped to LTR, LINE, SINE, and DNA class of MEs. Subfamilies are arranged by the age from oldest to youngest [24]. LTR1 subfamily with the highest piRNA mapping density is labelled on the plot.

Among different ME classes, only LTR were significantly enriched for piRNAs relative to their abundance in the genome (Figure 4A). The LTR subfamily showed the highest piRNA association is the LTR1/HUERS-P2 elements: 20% of the piRNAs mapped to the antisense direction of LTRs mapped to these elements (Figure 4B). HUERS-P2 is a low-copy number (~50) primate-specific family of ERV element who potentially encodes gag-like sequences [23], and only account for 0.14% of human genomic LTR elements.

To determine the relationship between the age of ME subfamilies and piRNA mapping density, we compared the age rank of 360 ME subfamilies [24] with their piRNA mapping densities. DNA element subfamilies showed no significant correlation between their ages and piRNA densities (Spearman’s rho = 0.13, p = 0.31). LINE (Spearman’s rho = −0.45, p = 5.3×10^−06^) and SINE (Spearman’s rho = −0.89, p = 0) elements showed significant negative correlation between the age of the subfamily and piRNA density (Figure 4C). In contrast, the ages of LTR subfamilies showed significant positive correlation with piRNA densities (Spearman’s rho = 0.58, p <10^−99^), implying that younger elements are associated with higher piRNA expression level, and they are more likely to be targeted by piRNAs (Figure 4C).

### piRNA mapping pattern in ME consensus sequences

**Figure 5:**
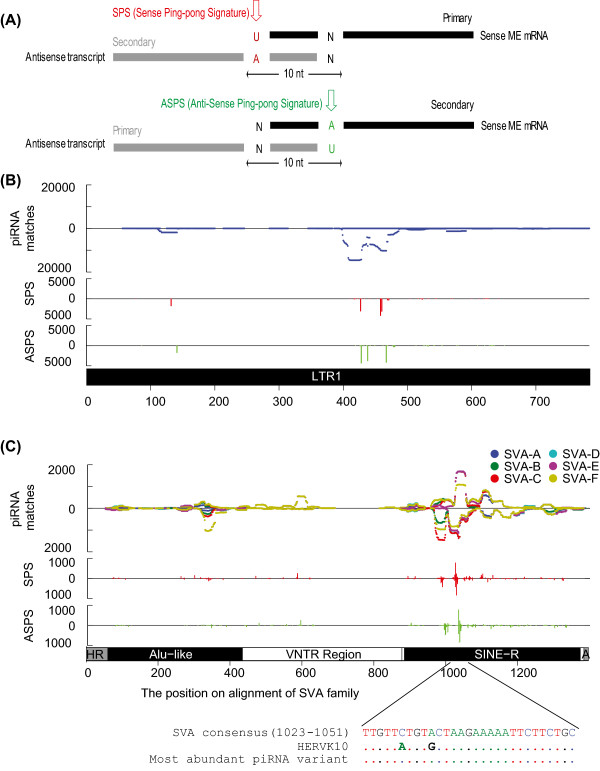
**piRNA mapping pattern in SVA and LTR1 elements. (A)** Two ping-pong models. Based on ping-pong models proposed in mouse and Drosophila, two types of ping-pong signature are examined: sense ping-pong signature (SPS) and antisense ping-ping signature (ASPS). piRNAs mapped to the sense and antisense strand of an ME are shown as black and grey solid boxes, respectively. Red and green arrows indicate the diagnostic base for SPS and ASPS, respectively. **(B)** piRNA mapping pattern in LTR1 element. Top subplot shows density of piRNA matches to the consensus sequence of LTR1. The second and third row present mapping densities of piRNAs exhibit SPS (red bars) or ASPS (green bars), respectively. Peaks above and below the X-axis indicates piRNAs mapped to sense and antisense of the ME consensus, respectively. A diagram of the LTR1 consensus is shown at the bottom of the plot. **(C)** piRNA mapping pattern in SVA element. Top subplot shows density of piRNA matches to the consensus sequences of different SVA subfamilies (SVA_A-F). The second and third row present piRNAs exhibit SPS (red bars) or ASPS (green bars), respectively. Peaks above and below the X-axis indicates piRNAs mapped to sense and antisense of the ME consensus, respectively. A diagram of the SVA consensus is shown at the bottom of the plot. To demonstrate that the piRNAs match SVA-specific sequences, the sequence alignment was shown for SVA consensus, HERVK10 consensus, and the most abundant piRNA sequence. HERVK10 specific mutations are shown as bold letter in the alignment.

To determine if piRNAs participate in ME regulation via the ping-pong cycle mechanism in human, we mapped piRNAs to the consensus sequences of major human ME subfamilies and analyzed the density, direction (sense or antisense), and potential ping-pong signatures of these piRNAs. If a ping-pong cycle mechanism is used for ME regulation, we expect the piRNAs to be processed from both sense and antisense strands of an ME [7, 8]. piRNAs that originated from the ME transcripts and targeting the ME transcripts should also show specific pairing patterns. Figure 5A illustrates the two expected patterns for piRNA pairs participating in the ping-pong cycle. According to the sequence signature at the first and the tenth bases of a piRNA, we divided the piRNAs matching the ping-pong cycle signature into sense ping-pong signature (SPS) or anti-sense ping-pong signature (ASPS) (Figure 5A). A piRNA is considered to have the SPS if it has an U at the first position (1U) and matches the sense strand of the ME consensus sequence, or it has an “A” at the 10^th^ position (10A) and matches the antisense strand of the ME consensus. On the other hand, a piRNA is considered to have the ASPS if it has 1U and matches the antisense strand or has 10A and matches the sense strand.We first investigated the piRNAs that mapped to LTR1 (Figure 5B) and SVA elements (Figure 5C). Most piRNAs mapped to the antisense strand of the LTR1 element between 400–500 bp of the consensus sequence. Six putative ping-pong signature peaks that contain more than 3,000 piRNAs could be detected on the antisense strand in this region, including three SPS and three ASPS peaks (Figure 5B). One region displays both SPS and ASPS signature (SPS 458 bp, ASPS 467 bp). Together these data suggest that LTR1/HUERS-P2 family of LTR element might be subject to an active piRNA ping-pong cycle in human testis.For SVA elements, the mapping patterns were largely congruent among the six subfamilies (Figure 5C). SVA elements are composed of three main sections, including a SINE-R section that is derived from HERV-K (human endogenous retrovirus family K). The SINE-R section in SVA overlaps partial internal region (886–993 bp, pol/env ORF) and partial 3’ LTR region (948–1276 bp, U3 region) of the HERV-K element. piRNAs primarily mapped to the SINE-R region of SVA around position 1000 bp on both strands and both SPS and ASPS peaks were observed (Figure 5C). To determine if this enrichment was due to the mapping of HERV-K derived piRNAs, we compared the SVA consensus sequence at the piRNA enriched region (1023–1051 bp) with the HERV-K consensus sequence. Within this region, the SVA and HERV-K consensus sequence differ by two nucleotide positions (Figure 5C, alignment). Therefore, it is unlikely that the HERV-K derived piRNAs will map to the SVA consensus and the piRNAs mapping to this region appear to be SVA-specific.

For Alu elements, we constructed three composite consensus sequences (AluJ, AluS, AluY), corresponding to the three major Alu lineages [25]. piRNAs showed distinct mapping patterns to the Alu consensus sequences. Several regions, including the RNA polymerase III internal promoter region at the 5’ end and middle A-linker region, showed a higher density of piRNA mapping (Additional file 1: Figure S8A). One SPS and one ASPS peak (>1,000 piRNAs) could be observed in the middle A-linker region, both on the sense strand and the piRNAs within the peak showed the highest similarity to AluY consensus (Additional file 1: Figure S8A). For L1, we constructed six composite consensus sequences (L1HS, L1P1-L1P4, and L1PB) corresponding to the major subfamilies defined by [26]. The 5’ end of the L1 consensus sequences, especially the 5’UTR region (~900 – 1,000 bp), showed higher mapping density (Additional file 1: Figure S8B). Unlike other ME families, no strong ping-pong signature peak can be identified on the L1 consensus, despite the fact that piRNA peaks are present on both strands of the consensus.

### Origin of piRNAs on the antisense strand of MEs

The most abundant piRNA peaks we observed within MEs were on the antisense direction of LTR1 and SVA consensus sequences. Next we determined if these piRNAs were transcribed from distinct piRNA clusters in the genome. To do so, we identified the perfect mapping positions of the most abundant piRNA sequences within the peak regions (see Methods for detail). Within LTR1, using piRNAs that mapped uniquely to one genomic location, we identified two piRNA clusters defined in our study: chr14:88,591,001-88,660,987 and chr21:45,873,525-45,923,566. About 98% of piRNAs that were mapped antisense to LTR1 can be mapped perfectly to these two clusters. The LTR1s in each of the two clusters exist as parts of a full-length HUERS-P2 element. The piRNA sequences were predicted to originate from three different LTRs (one 5’ LTR and two 3’ LTRs) from these two elements (Additional file 1: Figure S9A-B). Similarly, we identified one piRNA cluster (chr15:62,457,066-62,598,010) which produced most piRNAs mapping antisense to the SVA consensus (Additional file 1: Figure S9C-D). The putative SVA antisense cluster contains an SVA element belonging to the SVA-C subfamily. All three clusters were also presented in the piRNA clusters defined by [1].

## Discussion

In this study, we used an established periodate treatment and β-elimination protocol followed by Illumina high-throughput sequencing technology to analyze the piRNA population produced in three human adult testis samples. The putative piRNAs in our dataset showed characteristics of canonical piRNAs: they have a strong 1U bias, most of them fall in genomic clusters with strong directionality, and their overall genomic distribution closely resembles that from a previous study [1] (Additional file 1: Figure S2). All the evidence support that the vast majority of the sequences in our dataset are authentic piRNAs. Our data increases the total number of putative human piRNAs by more than two orders of magnitude. This larger data set allowed us to reveal several interesting new insights into piRNA function and evolution in the human lineage.

Our first contribution is a significantly improved annotation of piRNAs and piRNA clusters in the human genome. Our deeper sequencing over previous studies of human piRNAs (~17 million vs. ~50,000 in [1]) allowed us not only to recover the previously known ~180 piRNA clusters but also to identify up to 6,000 additional piRNA cluster candidates with generally lower but consistent expression signals in the three individual testis samples. Furthermore, this large data set provides us with increased statistical power to study in more detail the relationship of piRNAs with protein-coding genes, lncRNAs and mobile genetic elements. We realize that some of the piRNA cluster candidates with lower expression level could be false positives. Therefore, we provided the full list of the piRNA cluster candidates along with their expression level. Researchers who wish to increase the accuracy could apply more stringent expression level cutoff to the dataset.

To gain a better understanding of the primary piRNA biogenesis mechanism and the role of piRNAs in cellular gene regulation, we examined the piRNAs derived from protein-coding genes. One potential role for genic piRNAs is to reduce the expression level of the corresponding gene [12], while another potential regulatory role is to repress other genes *in trans* by pairing to mRNA with complementary sequence to the piRNA in a fashion similar to microRNAs [12]. Genic piRNAs could also direct chromatin modifications on the locus from which they are transcribed [27]. We found that exonic piRNAs are preferentially derived from the 3’UTRs of genes, especially genes with long 3’UTRs, similar to the findings in mouse and Drosophila [11]. It is not yet clear what accounts for the preference for 3’UTR piRNAs. Possibly the biogenesis machinery competes with the RNA translation machinery or the base composition of 3’UTRs makes these piRNAs more stable. Alternatively, the piRNA machinery could be guided to 3’UTRs because of interaction with protein(s) binding to 3’UTRs and/or coupling to mRNA splicing and mRNA decay pathways. Importantly, we found that genes enriched for 3’UTR piRNAs tend to be conserved between human and mouse, but not between these mammals and Drosophila. In addition, we discovered an enrichment of genes within function categories such as chromatin modifiers among genes that have high piRNA production level (Additional file 2: Table S4). Because one of the mechanisms piRNAs use to repress their targets is through DNA methylation, which is associated with chromatin remodeling and modification [28, 29], this functional enrichment is reminiscent of the dual roles of the Traffic Jam gene in the Drosophila piRNA pathway [12]: Traffic Jam protein activates Piwi expression and the Traffic Jam transcript is processed into piRNAs. It would be interesting to see if other mRNAs that are processed into piRNAs also code for proteins that play a role in the piRNA pathway. Taken together, these data suggest the potential biological importance of piRNAs in gene regulation.

Third, we examined the overlap of piRNAs and annotated lncRNAs. For a small set of lncRNAs (0.6%-3.2%), we observed significant overlap between putative piRNAs and the exons of lncRNAs on the same strand (Table 2). This small number of lncRNAs nonetheless accounted for up to 16% of all putative piRNAs (>900 fold enrichment). This result suggests that a small subset of currently annotated lncRNA genes might act as precursors for piRNAs. This does not preclude other functions for these lncRNAs since dual-function non-coding RNAs are known to exist: for example, tRNAs could also give rise to piRNAs. Consistent with the idea that some lncRNAs may enter the piRNA biogenesis pathway, it has been reported that a large fraction (one third) of human lncRNAs are testis-specific [30]. In addition, we also observed an enrichment of LTR/ERV class of MEs in lncRNAs enriched for piRNAs. Given the over-representation of LTR-associated piRNAs in our dataset, LTR-derived lncRNAs might be a prominent source of piRNAs in human testes.

Lastly, we were able to detect novel patterns in how piRNAs map to and presumably target mobile elements. When mapped to the consensus sequences of MEs, piRNAs show distinct mapping patterns, with some piRNA mapping peaks showing a signature of the ping-pong amplification cycle (Figure 5). This result suggests the piRNAs are not just randomly generated but are rather derived from or target specific regions of the MEs. However, most peaks in Alu and L1 consensus sequences have relatively low density (<2,000 piRNAs per peak), despite that Alu and L1 have the highest known retrotransposition activity in humans. The low piRNA mapping density and the rarity of ping-pong signatures in Alu and L1 MEs suggest that the ping-pong mechanism is not the primary mechanism for regulating ME activity in adult human testis, consistent with previous observations in mouse [28, 31, 32]. Nevertheless, the current data does not allow us to preclude a more important role for fetal testis or ovarian piRNAs in controlling these elements in the human germline.

In contrast, several observations suggest that adult testis piRNAs might be involved in regulating LTR/ERV activity in human. First, piRNA density within an LTR family is strongly correlated with the age of the family: younger LTR families have higher piRNA densities (Spearman’s rho = 0.58, p <10^−99^). We observe the opposite trend for SINEs and LINEs: older families have higher piRNA density. Second, LTR-associated piRNAs have the highest expression level among ME-associated piRNAs, and piRNA density in LTR elements is significantly higher than expected by chance (Figure 4A). In contrast, SINE and LINE families show lower than expected piRNA densities in the genome. Third, within LTR1 element, the ME family producing the highest number of piRNAs in our dataset, piRNAs are primarily derived from the antisense direction and enriched in several peak regions, suggesting the antisense piRNAs might recognize the LTR transcripts. It is known that LTR elements harbor promoter regions and could initiate transcription at their insertion sites, both for their own genes, as well as for adjacent cellular genes [22, 33]. piRNAs might be involved in targeting LTR transcripts and preventing adjacent host gene transcription.

LTR1 element was identified as the LTR of HUERS-P2 element by Harada et al. in 1987 [23], but little research has been done on this element since then. We tried to determine the structure of the LTR1 element by sequence features, but only TATA box at nucleotide position 175 (usually in the U3 region of an LTR) and polyadenylation signal at position 425 (usually in the U3 or R region) could be predicted in the LTR1 consensus sequence. Using TSS-seq dataset from human adult testis [34], we found the majority of TSSs mapped between nucleotide position 200 and 300 (Additional file 1: Figure S10). Therefore, the U3-R boundary might locate within this region. LTR1 mapped piRNAs are enriched between position 400 and 500 (Figure 5B) and do not overlap the TSSs, suggesting that they have a potential role of targeting LTR1 transcripts rather than disrupting transcription factor binding.

## Conclusions

Overall, our study provides the most comprehensive analysis of piRNAs from adult human testis to date. We note that although a few other small-scale studies on human total small RNAs have been recently reported [35, 36], they did not go in depth into piRNA analysis. Our analysis defines a catalog of human piRNA cluster candidates and sheds new light into the relationship between piRNAs and protein-coding genes, lncRNA genes, as well as mobile elements. These findings establish a foundation for future analyses of the function and evolution of piRNAs in human.

## Methods

### Sample preparation

We obtained two samples of total RNA from human testes from Ambion (cat no.AM7972). The RNA samples were extracted from autopsy tissues and do not require IRB approval. We extracted one additional RNA sample from frozen testis tissue obtained from National Disease Research Interchange (http://www.ndri.org). The testis tissue was collected from autopsied human remain and does not constitute human subjects as defined by federal regulations (45 CFR 46) and does not require IRB approval. The RNA extraction was performed using Trizol (Invitrogen) according to the manufacturer’s recommendations. All three individuals were Caucasian with ages ranging from 34 to 59 years old. We measured the RNA concentration for each sample with Nanodrop and the RNA Integrity Number (RIN) with the Agilent 2100 Bioanalyzer. There is no apparent degradation of the samples and we did not observe any systematic differences between the samples from Ambion and NDRI.

### Periodate oxidation and β-elimination treatment

For each sample, we subjected three micrograms (μg) of total testis RNA to PO treatment [37]. The PO treatment has been shown to be effective in separating piRNAs from other classes of small RNAs and degradation products of longer mRNA transcripts studies [6, 14, 15]. A 50 μl mixture consisting of 3 μg of total RNA and 10 mM NaIO_4_ was incubated at 0°C for 40 min in the dark, then 5 μl of 1 M rhamnose was added to quench the unreacted NaIO_4_ and incubated at 0°C for additional 30 min. Fifty-five μl of 2 M Lys-HCl (pH 8.5) was then added and the solution was incubated at 45°C for 90 min for β-elimination. The treated RNAs were purified using a standard ethanol precipitation protocol.

### piRNA sequencing library construction

We constructed piRNA sequencing libraries from the treated total RNAs using the NEBNext® Multiplex Small RNA Library Prep Set for Illumina® (New England Biolabs) following the manufacturer’s instructions. Briefly, the RNAs were ligated with 3’ and 5’ adaptors and converted to cDNA by reverse transcription. PCRs were then performed using different index primers for different individuals. The PCR conditions were: an initial step at 94°C for 30 sec, 12 cycles at 94°C for 15 sec, 62°C for 12 sec and finally 70°C for 15 sec. The amplified libraries were electrophoresed through a 3% NuSieve GTG (Lonza) 3:1 GenePure LE agarose gel (Bioexpress) and bands ~150 bp were excised. We purified the gel slices using the Wizard SV Gel and PCR Clean-up Kit (Promega). We validated the libraries for quantity and quality on a 2100 Bioanalyzer using a High Sensitivity DNA chip according to the manufacturer’s instructions. The libraries were then sequenced on an Illumina HiSeq 2000 machine using single-end 50 base-pair format. The sequences have been deposited to the NCBI short read archive (SRA) under study number [SRP021475].

### piRNA sequence processing and cluster identification

The raw sequencing reads were first computationally stripped off adapters using the Cutadapt tool [38] and reads that are between 5 and 45 bp after stripping were kept (Table 1, Step 1). We then aligned the reads to known small RNA genes downloaded from Ensembl (release 70, ftp://ftp.ensembl.org/pub/release-70/gtf/homo_sapiens/Homo_sapiens.GRCh37.70.gtf.gz), and RNA repeats from RepeatMasker human consensus sequences (repeatmaskerlibraries-20120418) using Bowtie [39] (version 0.12.8) allowing up to 1 mismatch ([−k 1 -v 1]). Reads mapped to known non-coding RNA genes (miRNAs, pre-miRNAs, rRNAs, scRNAs, snRNAs, snoRNAs, srpRNAs, tRNAs, MT_tRNAs, MT_rRNAs in Ensembl, and RNA repeats in RepeatMasker) were removed from the datasets (Table 1, Step 2). We then mapped the remaining reads to the human reference genome (hg19) using Bowtie (version 0.12.8) allowing up to 1 mismatch and multiple matches ([-a --best --strata -v 1]) (Table 1, Step 3). Reads mapped to the assembled chromosomes in the reference genome that are between 26–31 bp were selected as putative piRNAs (Table 1, Step 4).

piRNA clusters were identified from putative piRNAs using a procedure essentially identical to previously published methods [1, 18]. Specifically, we slid a 5 kb window by 1 kb steps along each chromosome and counted the normalized number of piRNAs in each window using the RPKM metric. Our RPKM definition is slightly different from the conventional RPKM definition. Because we do not have piRNA precursor transcript info, our RPKM is normalized on the genomic DNA length rather than the transcript length. Any window that met a minimum RPKM cutoff was considered a piRNA cluster and adjacent clusters were collapsed into one cluster. We analyzed piRNA clusters when allowing multiple mapped piRNAs or only using unique mapped piRNAs and did not see a large difference between these two sets because most piRNAs were uniquely mapped to the genome. Therefore we included piRNAs that mapped to multiple positions in the genome, but divided the number of such multiple-mapping piRNA reads by the number of mapping positions when estimating their relative abundance (e.g., a piRNA mapped to 10 positions was counted as 0.1 reads at each position).

### Normalized piRNA enrichment and gene transcript enrichment analysis

Genic piRNAs are defined as those that mapped to exonic regions of mRNAs on the sense strand, that is, 5’UTR, CDS, or 3’UTR based on RefSeq gene annotations (release number 56, NCBI, records start with “NM”). The number of piRNAs was normalized by the number of mapping positions. Then the normalized piRNA enrichment in each genic region was defined as follows: ((number of piRNAs in a region)/(number of piRNAs in a gene))/((length of a region)/(length of a gene)). For any gene with multiple isoforms, we selected the transcript with the highest number of mapped piRNAs. A gene is defined as “3’UTR enriched” if the normalized piRNA enrichment in 3’UTR was greater than that in CDS or that in 5’UTR. The GO analysis was performed using GO term finder [40].

### Conservation of piRNA producing genes

We mapped either mouse genes or Drosophila genes to human genes based on HomoloGene (release number 67, NCBI). When comparing the number of 3’UTR derived piRNAs in the homologous human genes to all other genes, we controlled for 3’UTR length, enrichment of piRNAs in 3’UTR, and gene expression level in testis to show the robustness of our result. Specifically, we compared 1) the number of piRNAs in a 3’UTR; 2) the number of piRNAs in a 3’UTR normalized by the length of the 3’UTR: (number of piRNAs in a 3’UTR) / (3’UTR length); 3) the normalized piRNA enrichment in a 3’UTR: ((number of piRNAs in the 3’UTR of a gene) / (number of piRNAs in the gene)) / ((length of a 3’UTR) / (length of a gene)); and 4) the number of 3’UTR piRNAs normalized by the gene expression level: (the number of piRNAs in a 3’UTR)/(gene expression level in testis).

### LncRNA analysis

Three resources were used for lncRNA loci annotation: 1) lncRNAs defined by Gencode ([41], release 15), 2) testis-expressed long intergenic non-coding RNAs from [30], and 3) testis-expressed long intergenic non-coding RNAs from [21]. The pooled lncRNA set consisted of 15,013 genes and 55,006 exons, occupying 19.2% and 0.85% of the human genome, respectively.

To calculate the piRNA expression level in lncRNA, we selected all piRNAs that were mapped to the sense strand of the exonic regions of lncRNAs. RPKM of piRNA expression in a feature (whole lncRNA, exonic region, non-exonic region) is calculated as (number of total normalized piRNAs in the feature) / (length of the feature in Kb) / (number of total normalized piRNAs in million). piRNA coverage of the exonic region of a lncRNA is calculated as (number of bases that piRNAs reside in the exonic regions) / (total length of the exonic regions).

### LncRNA mobile element analysis

Using BedTools [42], lncRNA exons and surroundings were joined (intersectBed with options wa and wb) with RepeatMasker output (hg19 assembly, RM v.330, repbase libraries 20120124, http://www.repeatmasker.org/species/homSap.html). Fragments with at least 10 bp of overlap were kept to calculate ME amount in exons. Non-ME elements (Low Complexity, Satellites, Simple Repeats and ncRNA) were not considered. Intersection files were then parsed using a custom perl script to (1) evaluate ME content and (2) determine the enrichment (or depletion) of each ME family relative to its genomic abundance.

1. ME content is defined by the intersection length of ME annotations and exon coordinates (corrected for overlaps between MEs). The counts of ME fragments are corrected using the interrupted repeats detection of RepeatMasker (for example, if a ME is fragmented in two because of a deletion or an insertion, it will be counted only one time. This correction does not account for inversions or complex rearrangements, but is more accurate than the basic fragment number).
2. For the analysis of over-represented MEs, 100% corresponds to the total amount (counts or length) of all different MEs in the analyzed set. For each ME, this proportion of counts or length is compared to the genomic abundance of that family (with 100% corresponding to the total amount of these same MEs in the genome). The ratio between counts (in-set divided by in-genome) was used to determine if a given ME was enriched or depleted. Significance of enrichment was inferred on ME counts with three standard statistical tests (binomial, hypergeometric, and Poisson models), the wordle plots (http://www.wordle.net/) were built on length since it is more representative of a given ME contribution.

### Genome-wide count of ME-derived piRNAs

The ME annotation of the human reference genome (hg19) was downloaded from the UCSC Genome Browser (http://hgdownload.cse.ucsc.edu/goldenPath/hg19/database/rmsk.txt.gz). A piRNA was considered overlapping with an ME if its mapping position is entirely within an ME. When calculating the number of piRNAs overlapping MEs in the genome, piRNAs that mapped to multiple locations were divided by the total number of mapping positions. For example, a piRNA that has 10 reported mapping positions will be counted as 0.1 piRNAs at any given position. After normalization, the total number of piRNAs mapped to a subfamily of ME was calculated.

### piRNA mapping signature in ME consensus

piRNA associated with MEs were identified using RepeatMasker version open-3.3.0 (http://www.repeatmasker.org) with customized consensus libraries. The custom ME libraries were constructed using consensus sequences of Alu, L1, SVA, and LTR subfamilies in the default library. The Alu subfamilies were divided into three groups, AluY, AluS and AluJ, corresponding to the three major Alu lineages [25] and L1 subfamilies were separated into six groups based on the major L1 lineages in primates, L1HS, L1P1, L1P2, L1P3, L1P4, and L1PB [26] (Additional file 2: Table S6). For each group, a multiple alignment was constructed using consensus sequences of all subfamilies within the group and a composite consensus was created allowing variation for each position. When insertion/deletion polymorphisms are present in the alignment, the custom library for the subfamily included two consensus sequences: include-all-insertion consensus, where all insertions in the consensus alignment are included; and include-all-deletion consensus, where all deletions in the consensus alignment are included. In combination, the two consensus sequences provide consistent mapping position for piRNAs for each group. For the SVA analysis, the six SVA subfamily consensus sequences in the RepeatMasker library (SVA_A-SVA_F) were used. For the LTR analysis, because the vast majority of the piRNAs mapped to the LTR1 and HUERS-P2 element, only these two consensuses were used for the analysis.

After the positions of piRNA in ME consensus were determined, the sense and antisense direction of piRNA versus MEs were determined from the RepeatMakser output. The ping-pong cycle signature (10 bp overlap between sense and antisense piRNAs) were identified using the intersectBed function in Bedtools [42]. According to sequence signature at the first and the 10^th^ base, we divided the piRNAs matching the ping-pong cycle signature into sense ping-pong signature (SPS, 1U in sense or 10A in antisense strand) and antisense ping-pong signature (ASPS, 10A in sense or 1U in antisense strand), respectively. The plots of piRNAs mapped to MEs were generated using Matlab.

To identify putative piRNA clusters in the genome where the antisense piRNAs were expressed, genomic locations of piRNAs within the highest antisense peaks of the LTR1 and SVA consensus sequences were determined. BLAT (The BLAST-Like Alignment Tool) [43] was used to identify the genomic position of each piRNA, allowing no mismatch. The putative piRNA cluster position was determined using uniquely mapped piRNAs.

## Electronic supplementary material

Additional file 1: Figure S1: Size distribution of sequence reads in a piRNA sequencing library. **Figure S2.** Properties of piRNAs within piRNA clusters. **Figure S3.** 688 homologous human genes (HHG) that are homologous to mouse 3’UTR piRNA enriched genes in mouse testis (10 dpp). **Figure S4.** 300 homologous human genes (HHG) that are homologous to mouse 3’UTR piRNA enriched genes in mouse adult testis. **Figure S5.** 51 homologous human genes (HHG) that are homologous to Drosophila 3’UTR piRNA enriched genes in Drosophila ovary somatic sheet (OSS) cells. **Figure S6.** Coverage of different ME classes in genome, lncRNA exons, and protein-coding gene exons. **Figure S7.** Wordle representation of ME abundance and enrichment in lncRNAs. **Figure S8.** piRNA mapping pattern in Alu and L1 elements. **Figure S9.** Origin of antisense piRNAs in LTR1 and SVA elements. **Figure S10.** Positions of TSS in LTR1 elements in human adult testis. (PDF 6 MB)

Additional file 2: Table S1: Number of piRNA clusters with different RPKM cutoffs. **Table S2.** Genomic coordinates of piRNA clusters with RPKM. **Table S3.** The percentage of 3’UTR enriched genes with different piRNA density cutoffs. **Table S4.** GO terms enriched in 3’UTR piRNA enriched genes, genes with the longest 3’UTRs, and genes with the highest expression in testis. **Table S5.** Putative piRNA-producing lncRNAs. **Table S6.** L1 group composition. (XLSX 549 KB)

## Abbreviations

MEs: Mobile elements
lncRNA: Long non-coding RNA
PO treatment: The periodate oxidation and β-elimination treatment
U: Uridine
RPKM: Reads per kilobase per million mapped piRNA reads
GO: Gene ontology
Mbp: Million base pair
TSS: Transcription start site
SPS: Sense ping-pong signature
ASPS: Anti-sense ping-pong signature
HERV-K: Human endogenous retrovirus family K
RIN: RNA integrity number
SRA: Short read archive
nt: Nucleotides.
