# Supplementary material for: A comprehensive analysis of piRNAs from adult human testis and their relationship with genes and mobile elements
Source: BMC Genomics. 2014 Jul 1;15(1):545. doi: 10.1186/1471-2164-15-545 (PMC4094622; doi:10.1186/1471-2164-15-545)
Supplement: Supplementary file 1 — Additional file 1: Figure S1: Size distribution of sequence reads in a piRNA sequencing library. Figure S2. Properties of piRNAs within piRNA clusters. Figure S3. 688 homologous human genes (HHG) that are homologous to mouse 3’UTR piRNA enriched genes in mouse testis (10 dpp). Figure S4. 300 homologous human genes (HHG) that are homologous to mouse 3’UTR piRNA enriched genes in mouse adult testis. Figure S5. 51 homologous human genes (HHG) that are homologous to Drosophila 3’UTR piRNA enriched genes in Drosophila ovary somatic sheet (OSS) cells. Figure S6. Coverage of different ME classes in genome, lncRNA exons, and protein-coding gene exons. Figure S7. Wordle representation of ME abundance and enrichment in lncRNAs. Figure S8. piRNA mapping pattern in Alu and L1 elements. Figure S9. Origin of antisense piRNAs in LTR1 and SVA elements. Figure S10. Positions of TSS in LTR1 elements in human adult testis. (PDF 6 MB) [file 12864_2014_6225_MOESM1_ESM.pdf]

A comprehensive analysis of piRNAs from adult human testis and their relationship with genes and mobile elements

Supplemental Materials

Hongseok Ha, Jimin Song, Shuoguo Wang, Aurélie Kapusta, Cédric Feschotte, Kevin C. Chen, and Jinchuan Xing

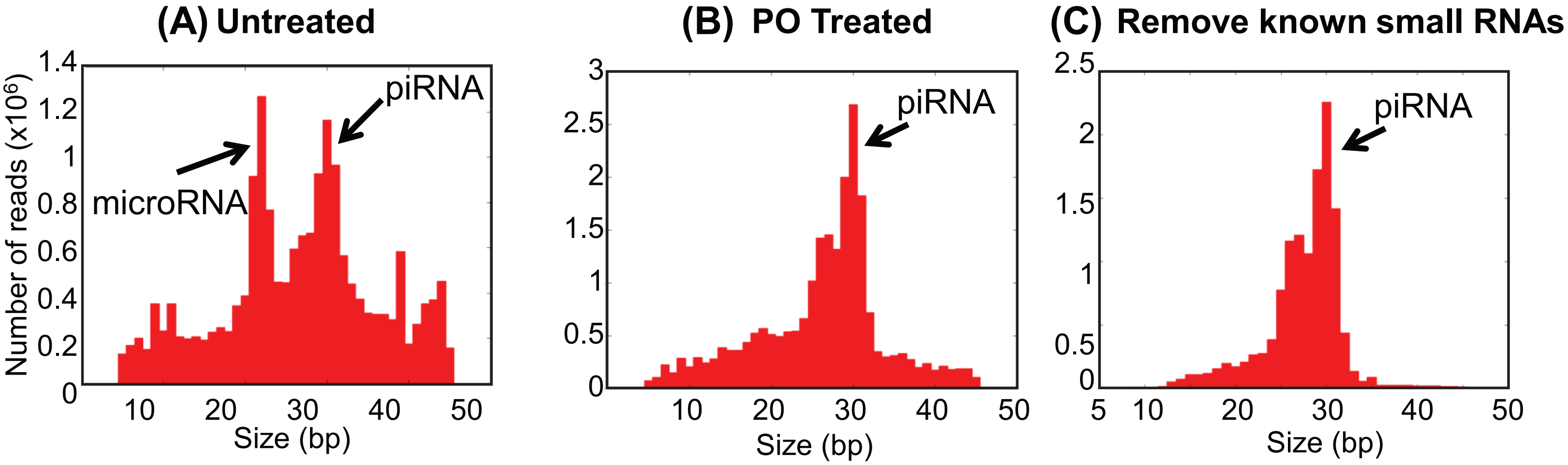

Figure S1. Size distribution of sequence reads in a piRNA sequencing library. **(A)** Without periodate oxidation and β-elimination treatment (PO treatment) **(B)** With PO treatment; and **(C)** With PO treatment, after removing other known small RNAs.

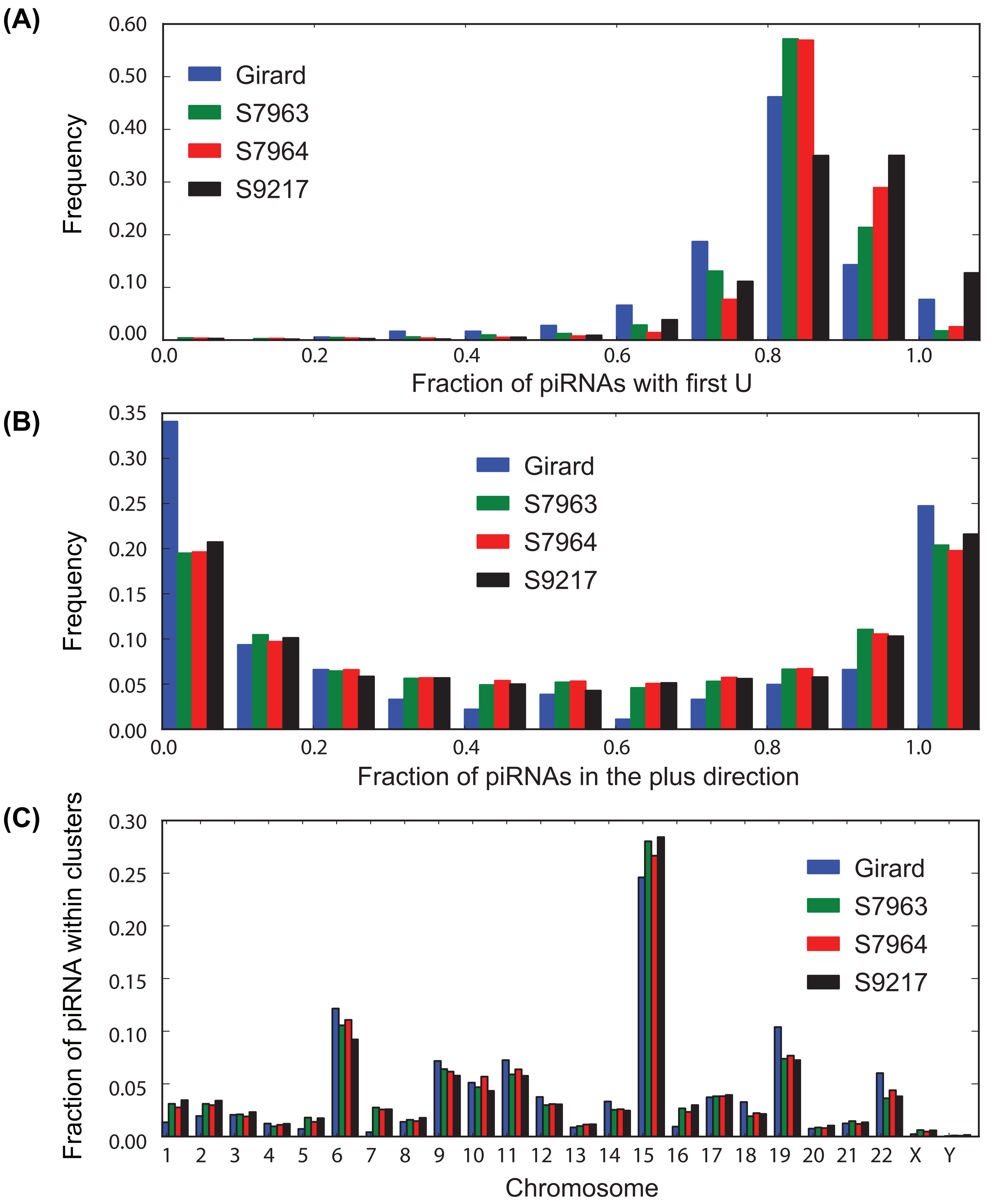

Figure S2. Properties of piRNAs within piRNA clusters.
Properties of piRNAs within the piRNA clusters identified in the three samples are compared with those from Girard et al. [1]. **(A)** Percentage of first U; **(B)** Percentage of unidirectional cluster; **(C)** Chromosomal distribution of piRNA clusters.

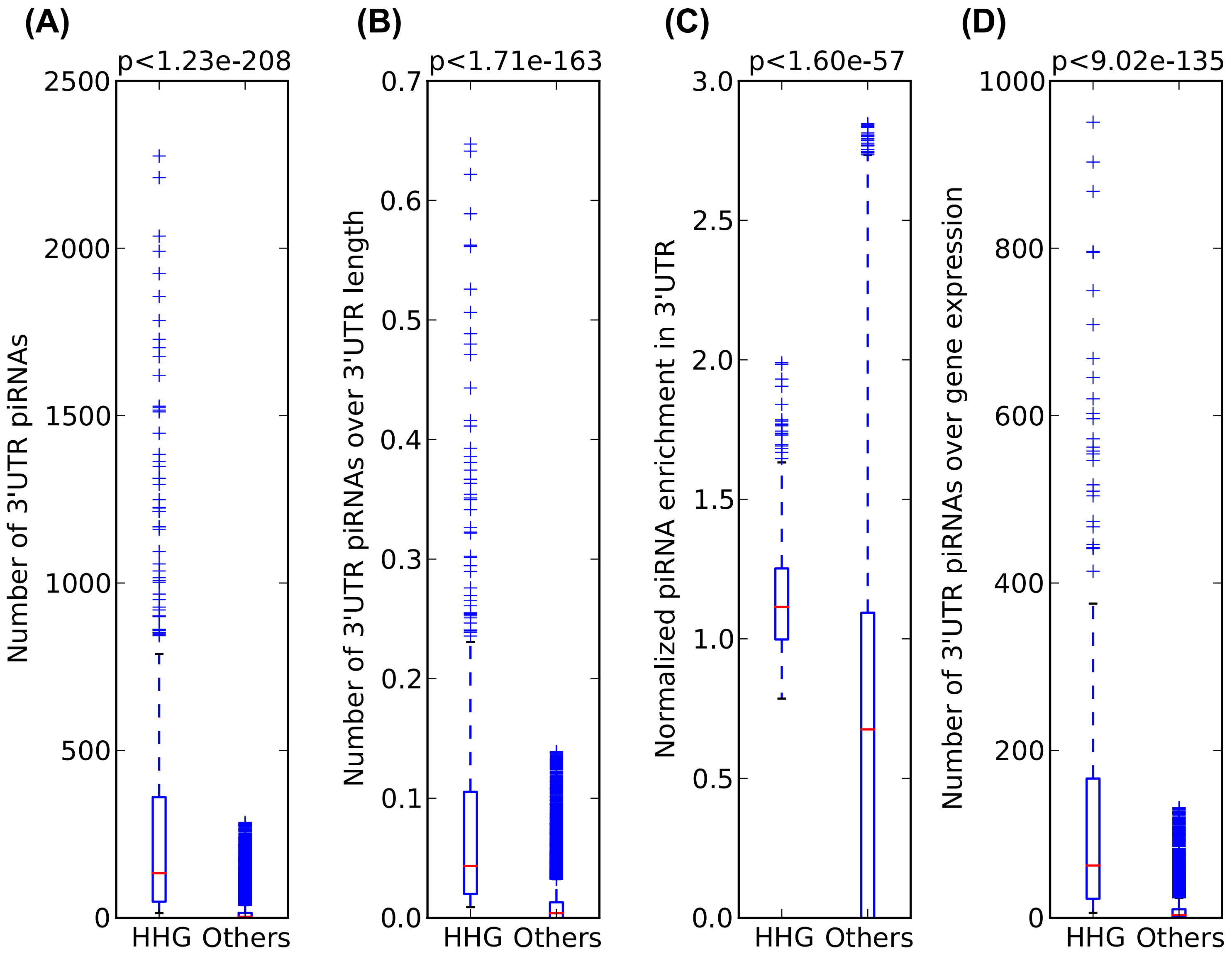

Figure S3. Number of 3'UTR piRNAs in human genes.
HHG: 688 human genes that are homologous to mouse 3'UTR piRNA enriched genes in mouse testis (10 dpp). Others: all other human genes. **(A)** box plots of the total number of 3'UTR piRNAs; **(B)** box plots of the total number of 3'UTR piRNAs normalized by the length of 3'UTRs (i.e., number of piRNAs in 3'UTR/3'UTR length); **(C)** box plots of the 3'UTR piRNAs enrichment in 3'UTRs (i.e., (number of piRNAs in the 3' UTR of a gene / number of piRNAs in the gene) / (length of a 3' UTR /length of a gene)); **(D)** box plots of the total number of 3'UTR piRNAs normalized by the gene expression level (i.e., number of piRNAs in a 3'UTR / gene expression level in testis). For visual purposes, genes within the 2nd and the 98th percentiles were shown.

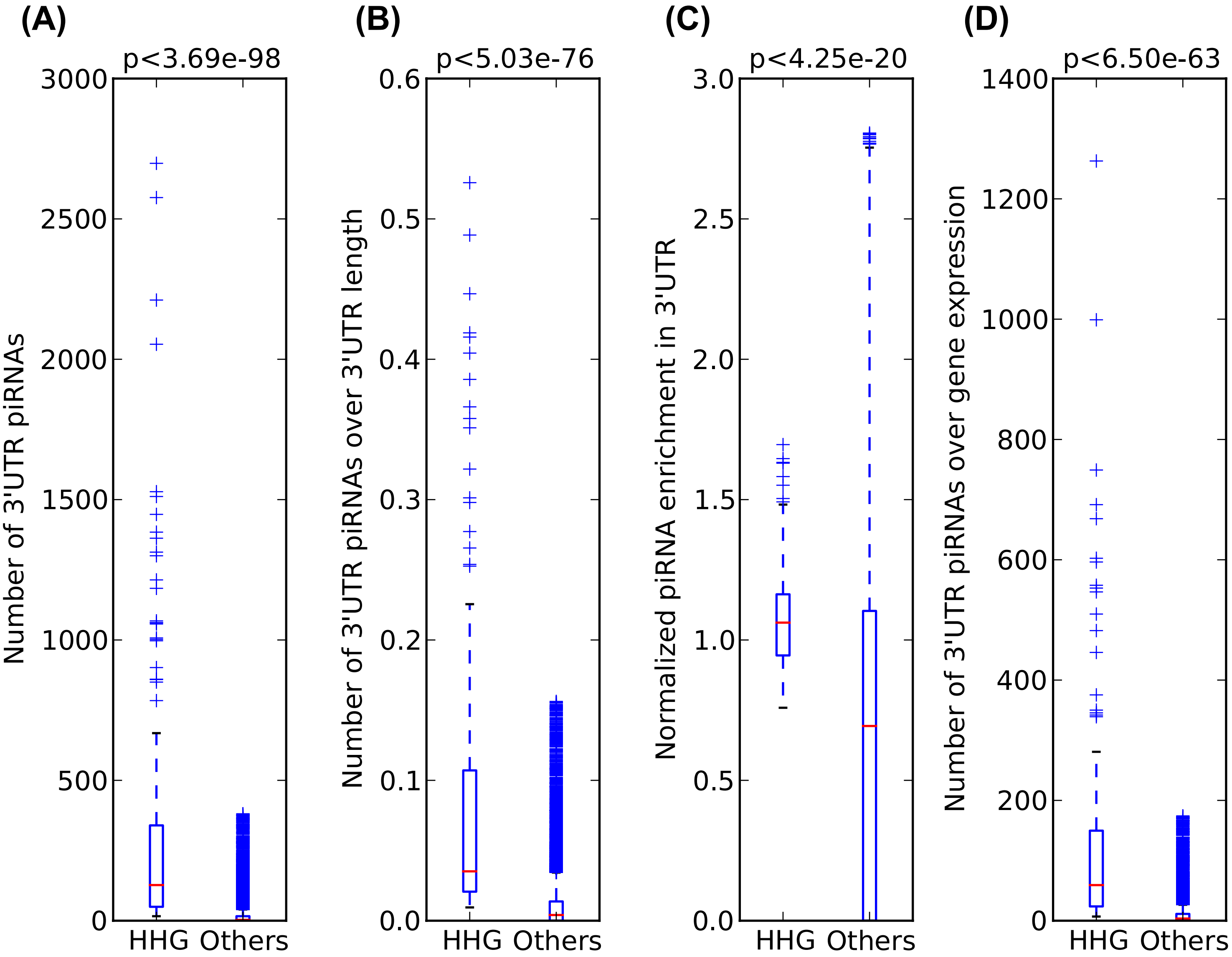

Figure S4. Number of 3'UTR piRNAs in human genes.
HHG: 300 human genes that are homologous to mouse 3'UTR piRNA enriched genes in mouse adult testis. Others: all other human genes. **(A)** box plots of the total number of 3'UTR piRNAs; **(B)** box plots of the total number of 3'UTR piRNAs normalized by the length of 3'UTRs (i.e., number of piRNAs in 3'UTR/3'UTR length); **(C)** box plots of the 3'UTR piRNAs enrichment in 3'UTRs (i.e., (number of piRNAs in the 3' UTR of a gene / number of piRNAs in the gene) / (length of a 3' UTR /length of a gene)); **(D)** box plots of the total number of 3'UTR piRNAs normalized by the gene expression level (i.e., number of piRNAs in a 3'UTR / gene expression level in testis). For visual purposes, genes within the 2nd and the 98th percentiles were shown.

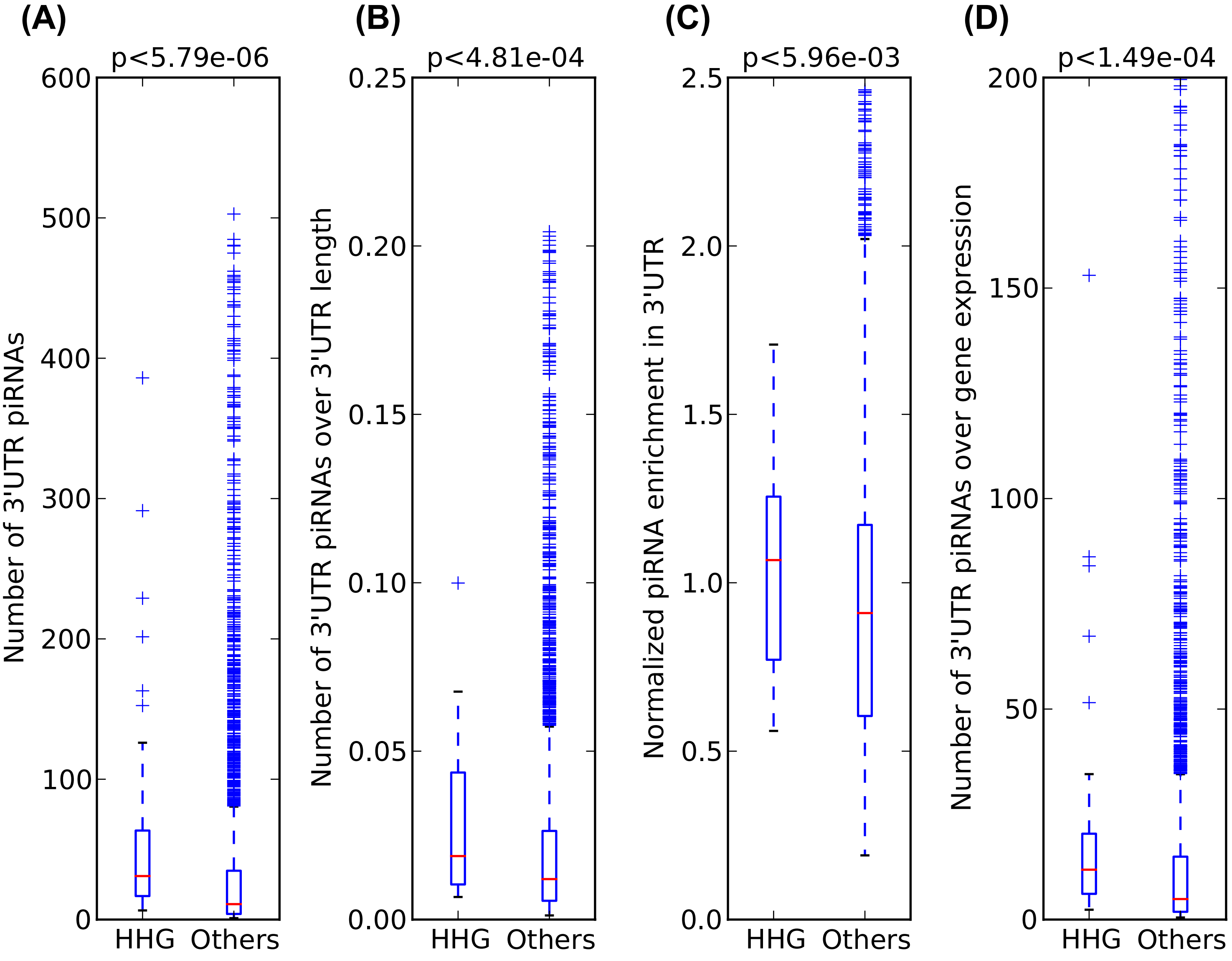

Figure S5. Number of 3'UTR piRNAs in human genes.
HHG: 51 human genes that are homologous to mouse 3'UTR piRNA enriched genes in Drosophila ovary somatic sheet (OSS) cells. Others: all other human genes. **(A)** box plots of the total number of 3'UTR piRNAs; **(B)** box plots of the total number of 3'UTR piRNAs normalized by the length of 3'UTRs (i.e., number of piRNAs in 3'UTR/3'UTR length); **(C)** box plots of the 3'UTR piRNAs enrichment in 3'UTRs (i.e., (number of piRNAs in the 3' UTR of a gene / number of piRNAs in the gene) / (length of a 3' UTR /length of a gene)); **(D)** box plots of the total number of 3'UTR piRNAs normalized by the gene expression level (i.e., number of piRNAs in a 3'UTR / gene expression level in testis). For visual purposes, genes within the 2nd and the 98th percentiles were shown.

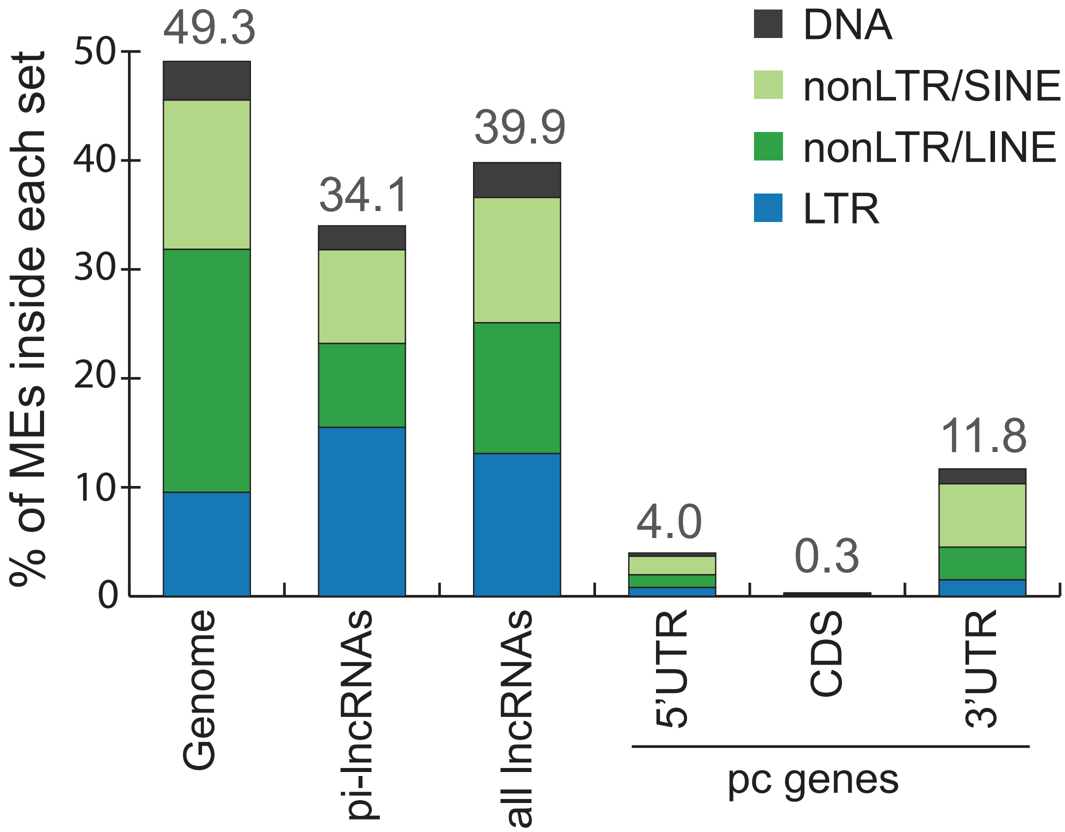

Figure S6. Coverage of different ME classes in genome, lncRNA, and protein coding exons. For human genomes, total length (100%) corresponds to total length of hg19 assembly without gaps (2,897 Mb). For the lncRNAs, and pi-lncRNAs, 100% corresponds to the total length of genomic projection of all the exons (27.1 Mb and 1.3Mb, respectively). For protein-coding genes (pc genes), total length of CDS, 5'UTR and 3'UTR exons are 3 0.9 Mb, 5.2 Mb, 24.6 Mb, respectively. Only pc genes from Refseq annotations with CDS and UTR features are considered. Percentage of coverage of all MEs is indicated above bars.

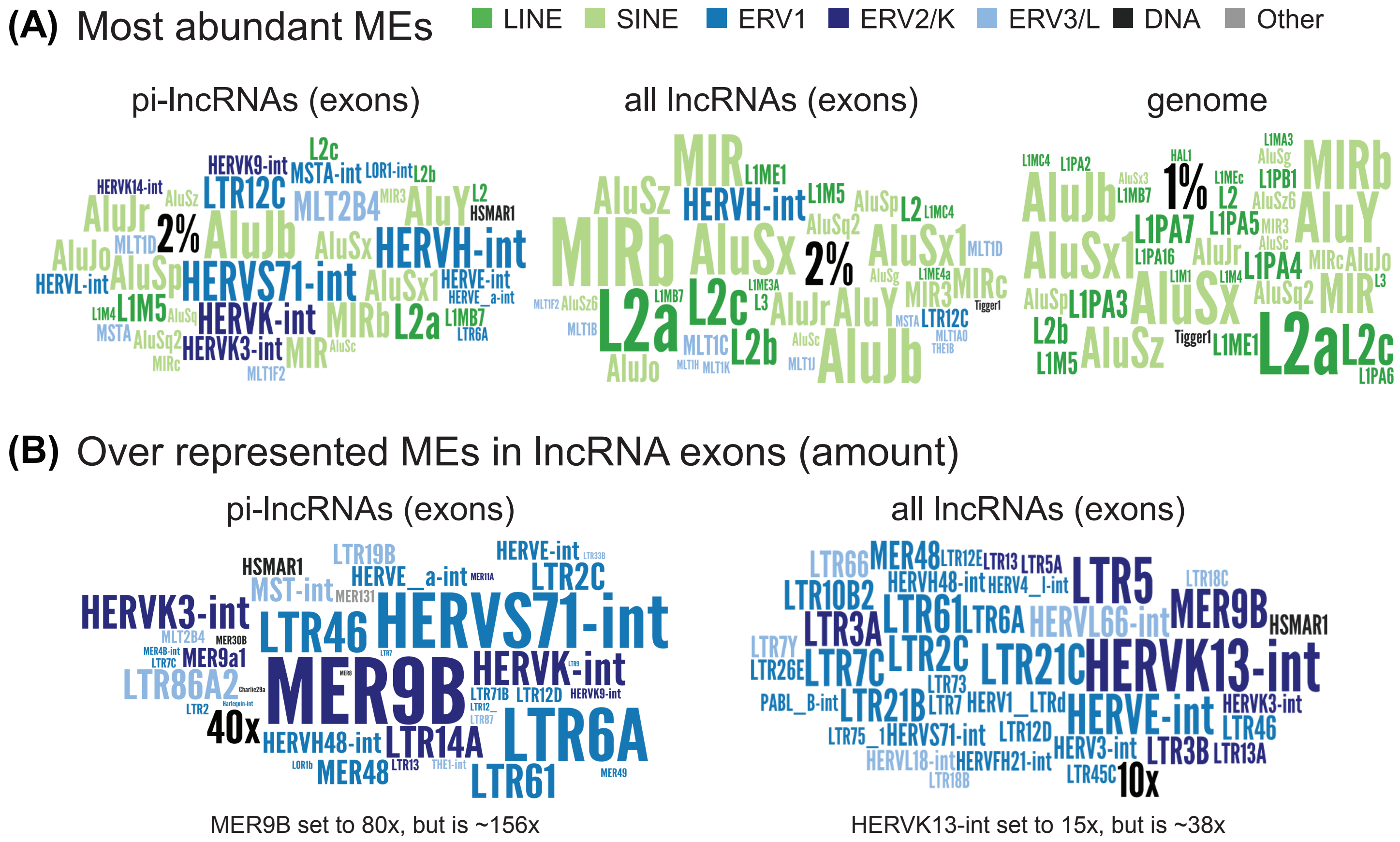

Figure S7. Wordle representation of ME abundance and enrichment in lncRNAs.
Colors refer to different ME classes: blue colors = LTR/ERV, green = LINE, light green = SINE, black = DNA, grey = unknown. **(A)** Visual representation of the 40 most abundant ME families in pi-lncRNAs, all lncRNAs and genome. Size of the ME family name is proportional to the percentage of ME derived DNA (scale of 1 or 2% in black). **(B)** Visual representation of the 40 most over-represented ME families in pi-lncRNAs and all lncRNAs, in exons. Size of the ME family name is proportional to its over-representation in terms of DNA amount (scales of 10x or 40x in black). The expected and observed counts of fragments corresponding to each ME are calculated using RepeatMasker output (see Methods). Observed values are obtained by considering overlapping MEs lncRNA exons (see Methods). Expected values are calculated b ased on the overall density of each ME family in the genome according to the RepeatMasker output assuming a random distribution of ME family members throughout the genome. Only f amilies statistically enriched in term of counts (fragment numbers) are kept (at least p-value<0.05, binomial distribution test). MEs with less than 2 fragments in lncRNAs are removed. Note that most over-represented MEs in pi-lncRNAs are low copy numbers.

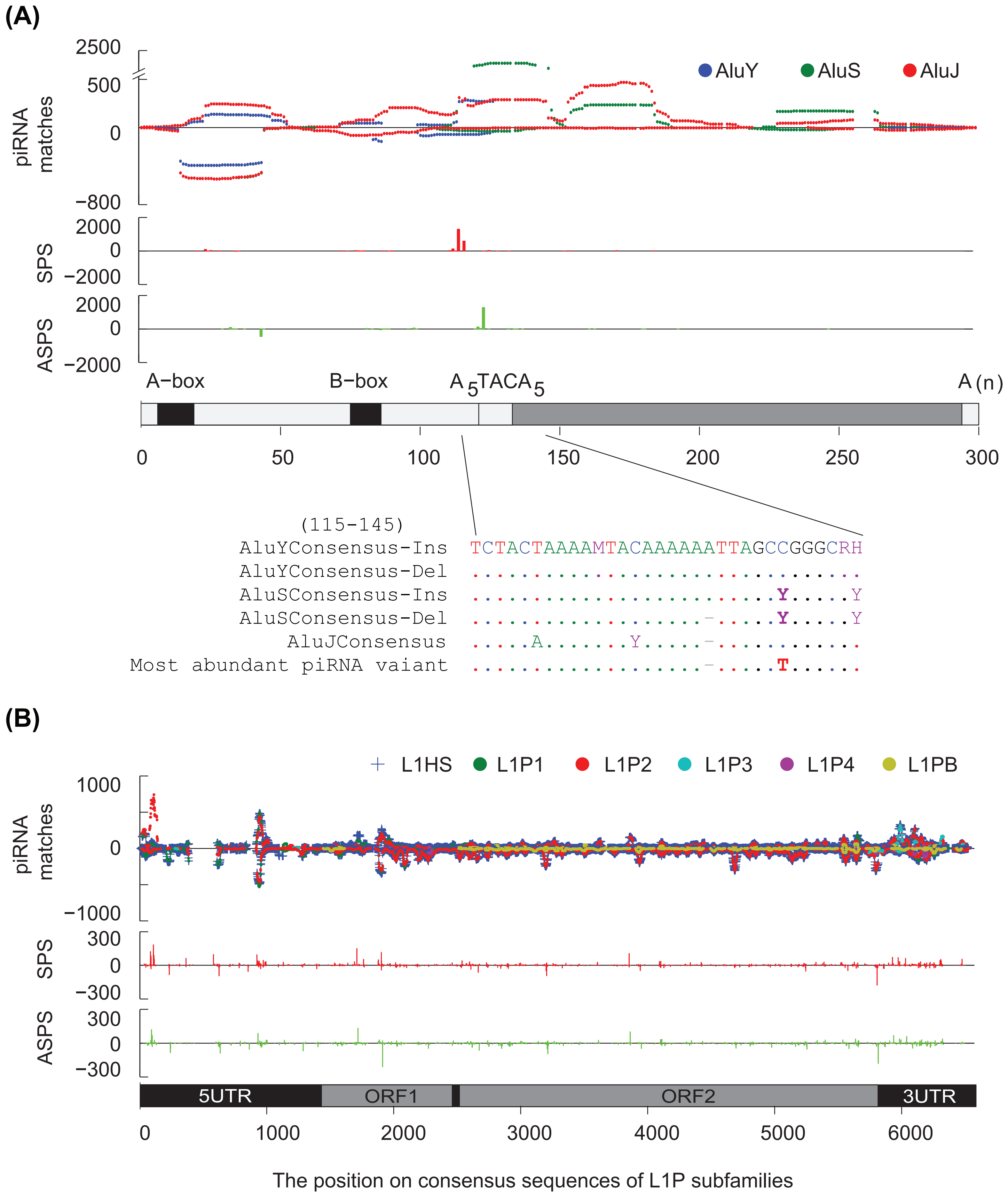

Figure S8. piRNA mapping pattern in Alu and L1 elements.

Figure S8 (continued) (A) piRNA mapping pattern in Alu element. Top subplot shows density of piRNA matches to the consensus sequences of different Alu subfamilies (AluY, AluS, AluJ). The second and third row present piRNAs exhibit SPS (red bars) or ASPS (green bars), respectively. A diagram of the Alu consensus is shown at the bottom of the plot. To demonstrate that the piRNAs match Alu-specific sequences, the sequence alignment was shown for all Alu subfamily consensuses and the most abundant piRNA sequence. Positions that are different from AluY c onsensus sequence are shown as bold letters in the alignment. **(B)** piRNA mapping pattern in L1 elements. Top subplot shows density of piRNA matches the consensus sequences of six L1 subfamilies. The second and third row present piRNAs exhibit SPS (red bars) or ASPS (green bars), respectively. A diagram of the L1 consensus is shown at the bottom of the plot.

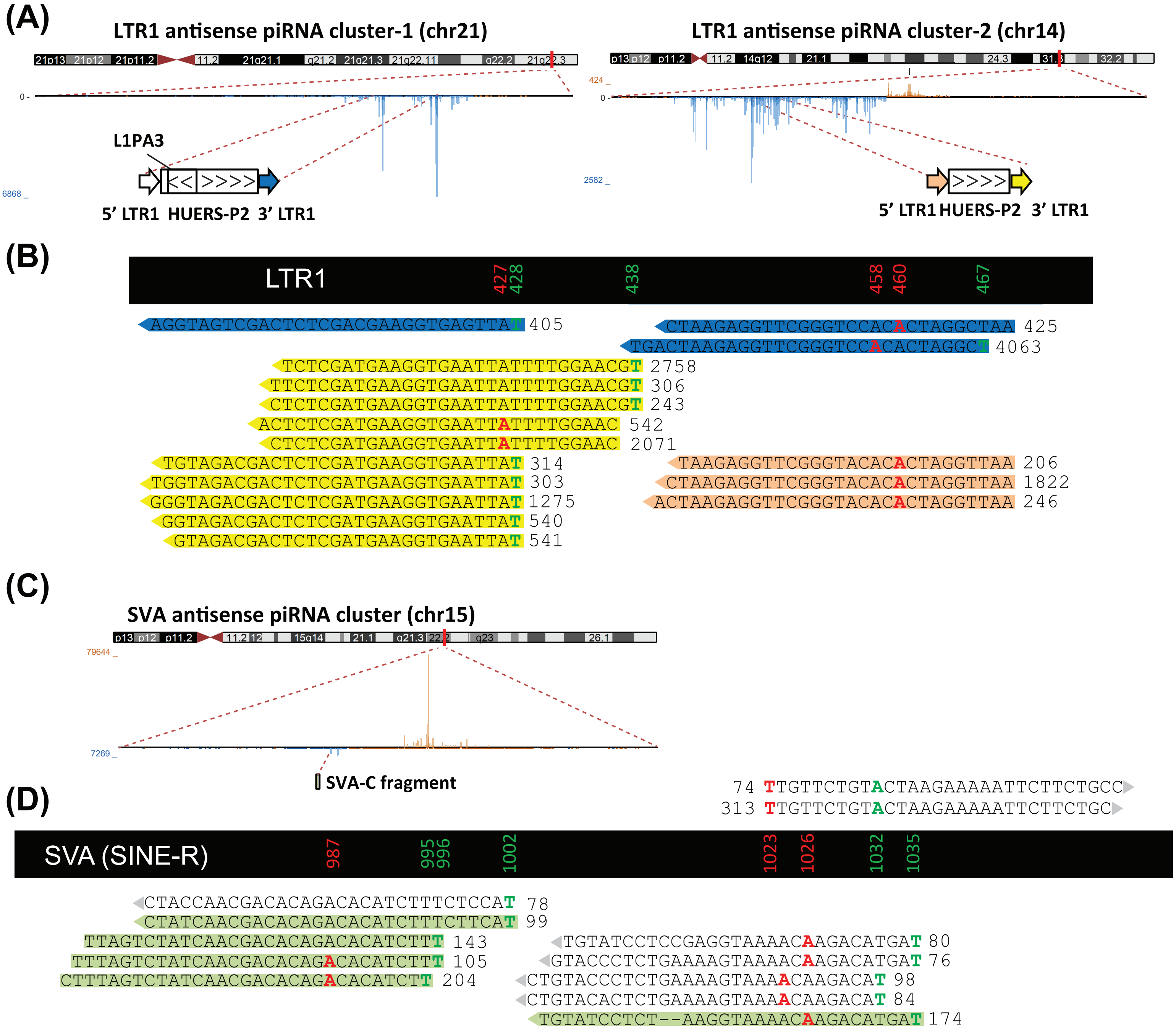

Figure S9. Origin of antisense piRNAs in LTR1 and SVA elements.
(A) The putative piRNA clusters expressing antisense LTR1 piRNAs. Two piRNA clusters on chromosome 21 and 14 are shown. The locations of LTR1 elements are illustrated and the thick arrows with different colors (blue, orange and yellow) indicate different LTR1 elements, respectively. **(B)** Major antisense piRNA variants with ping-pong signature in the region 400-500 bp of the LTR1 element. PiRNAs with more than 200 reads are presented under the LTR1 diagram and their putative origin is indicated by their colors corresponding to the colors of LTRs in (A). **(C)** The putative piRNA clusters expressing antisense SVA piRNAs. The piRNA cluster on chromosome 15 containing an SVA fragment is shown. The location of the SVA element is illustrated by a green box. **(D)** Major antisense piRNA variants with ping-pong signature in the region 950-1050 bp of the SVA element. PiRNAs with more than 70 reads are presented under the SVA diagram and piRNAs that are derived from the chromosome 15 cluster are shown in green.

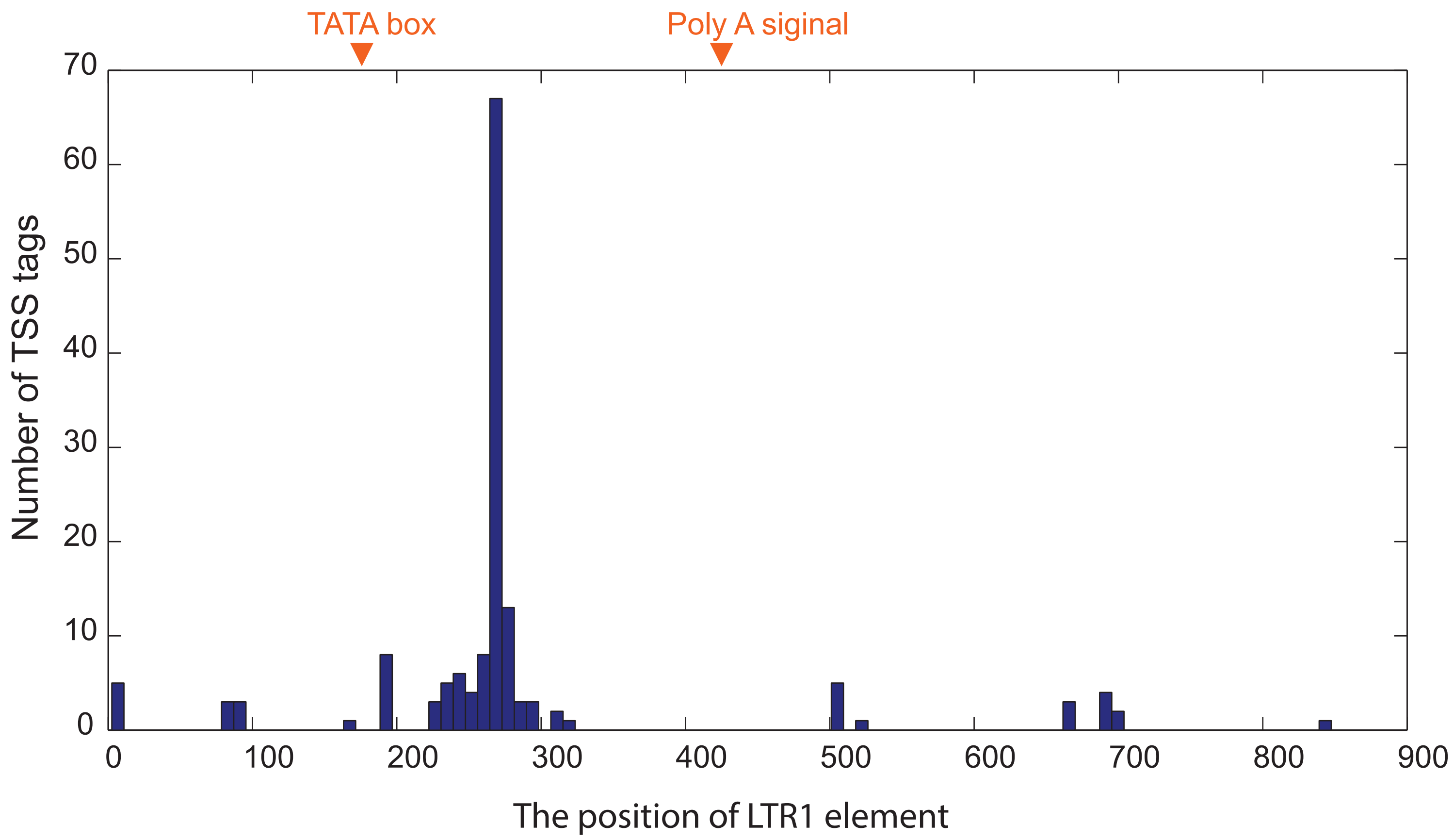

Figure S10. Positions of TSSs in LTR1 elements in human adult testis.
The mid-point position for each Transcription Start Site (TSS) tag is plotted. The putative positions of the TATA box and Poly A signal are indicated.
